# The influence of a dam reservoir on water quality in a small lowland river

**DOI:** 10.1007/s10661-021-08905-6

**Published:** 2021-02-15

**Authors:** Katarzyna Dębska, Beata Rutkowska, Wiesław Szulc

**Affiliations:** Institute of Agriculture, Department of Agriculture Chemistry, Warsaw, University of Life Sciences, Nowoursynowska 166, 02-787 Warsaw, Poland

**Keywords:** Biogenic compounds, Chemical oxygen demand, Dam reservoir, Water pollution

## Abstract

The paper presents the effects of the dam reservoir in Komorów on the water quality in the Utrata river. The implementation of the adopted objective involved a comparison of water quality at two points, above and below the reservoir. The Utrata River is polluted with biogenic compounds throughout the whole section studied. COD content also indicates significant contamination exceeding permissible limits. A positive effect of the reservoir on water quality in the river was also observed in terms of the content of dissolved oxygen, with concentration increasing below the reservoir. The reservoir had a positive effect on reducing the concentration of total phosphorus in the water. Water in the Utrata below the reservoir showed higher values of chemical oxygen demand (COD_Mn_) than above the reservoir. There were no differences in the concentration of NH_4_^+^ and NO_3_^-^ ions in the water before and after the reservoir.

## Introduction

Retention reservoirs are a very important element of water management and a source of drinking water. They contribute to the improvement of water conditions during draughts and reduce flood flows by retaining water (Wiatkowski, 2011a, 2011b). Lakes and retention reservoirs also affect the quality of the rivers flowing through them, and the magnitude of the effect depends on the depth, size, as well as cleanliness of the rivers flowing into the water reservoir (Kijowska – Strugała, 2016).

Catchment management and therefore proper use, reclamation and protection of lakes and retention reservoirs are keys for the preservation of the natural ecosystems of rivers and water for municipal and economic purposes (Krengel et al., 2018). Pollutants transported by overland flow from built-up areas are one of the causes of the worsening of water quality (Gnecco et al., 2005). In the case of catchments of lowland rivers, water quality is usually considerably affected by agriculture, contributing to the supply and increased concentration of nutrients in rivers. Total phosphorus and nitrates have a very negative effect on the water quality in rivers and lakes they flow through. Historically, the period in which waters were most threatened was spring, due to higher than average water flow, activating phosphorus deposited in the bottom sediments of the reservoirs and increasing supply from the catchment. Over the recent years, however, draughts in the spring periods have caused a change in these dynamics (Górski et al., 2019). International research shows that in Poland, a considerable part of the phosphorus load is supplied to flowing waters by agriculture (Mekonnen & Hoekstra, 2017). In recent years in Poland, pollution of flowing waters from point-based sources has been substantially reduced. Many wastewater treatment plants were constructed and modernised. Therefore, surface pollutants from agriculture constitute the primary source of nitrate nitrogen (Szalińska et al., 2018; Burzyńska, 2019).

The objective of the study was the analysis of the effect of the retention reservoir on changes in the values of selected water quality indicators in the Utrata River.

## Methods

### Description of the area study

**Fig. 1:**
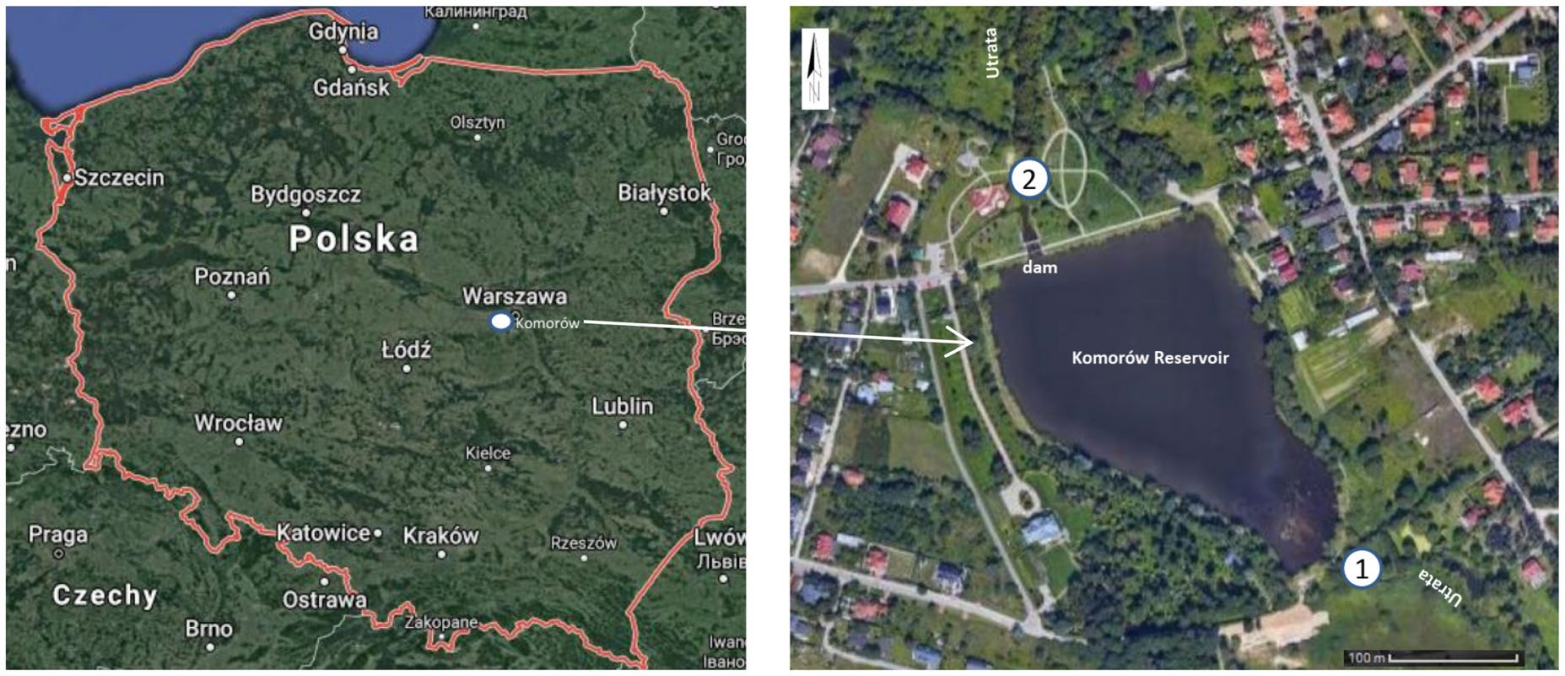
Study area—location control-measurement points 1 and 2 in the Komorów reservoir

The reservoir in Komorów is located in the Utrata River valley between the Komorów and Małe Pęcice villages, in the Pruszkowski poviat (Mazovian Voivodeship) 52°08′21.1″N 20°49′54.8″E (Fig. 1).

A mill functioned in the area of the modern reservoir, and water was accumulated there already before World War 2, although the retention reservoir itself was constructed in 1992. The surface area of the reservoir is approximately 6 ha. The Utrata River flows into the reservoir from the south and flows out to the north through a dam. The reservoir is located between Pęcice and Komorów and was formed through water damming. In addition to its retention purpose, the reservoir also fulfils recreational functions. A playground is located on its northern side. The reservoir in Komorów plays a very important role in the reduction of flood flow. The catchment area above the reservoir is 114.5 km^2^. Water reservoirs on the river contribute to limiting the load of pollutants flowing through it. The Utrata River, on which the analysed retention reservoir functions, is a right-bank tributary of the Bzura River to which it flows in Sochaczew, has a length of 78 km, and catchment area of 702 km^2^. The Utrata is exposed to pollution from the following sources: fish ponds, wastewater treatment plants, heat and power plants, waste dumps and progressing urbanisation. Another important source of pollutants supplied to the river is agriculture, which provides Warsaw with food. A considerable part of the catchment is occupied by Gleyic Phaeozems (WRB World reference base for soil resources 2014) (Kabała et al., 2019). Due to this, agriculture in the area is strongly developed and specialised (Wojtkowska, 2006). Water samples for physicochemical analyses were collected from the Utrata River above (control-measurement point 1) and below (control-measurement point 2) the reservoir every month throughout the hydrological year, i.e., in the period from April 2018 to March 2019.

### Chemical analysis

The following parameters were analysed (Fig. 1):

- pH according to norm PN-EN ISO 10523:^2012^.
- Temperature
- Dissolved oxygen according to the norm PN-EN 25813:^1997^
- Chemical oxygen demand according to the norm PN-EN ISO 8467:^2001^
- Total phosphorus according to the norm PN-EN ISO 6878:^2006^
- Ammonia nitrogen and nitrate nitrogen by the flow-through method according to the norm PN-EN ISO 11732:^2007^.

### Statistical analysis

For the statistical processing of the obtained results, a multifactorial analysis of variance (ANOVA) was applied. The determination of the significance of differences between mean values was employed using Tukey’s test, at a significance level of *p* = 0.05. The statistical analysis of results was performed with the application: Statistica ver. 10 software.

## Results

**Fig. 2:**
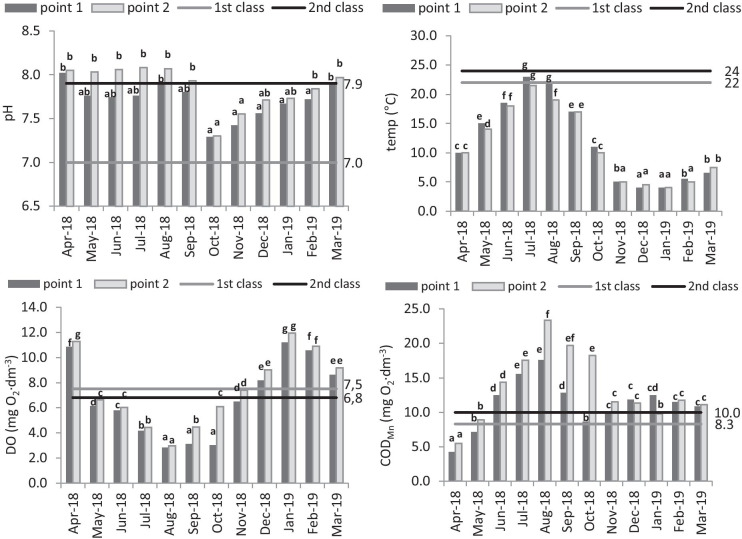
Seasonal variability of the pH, temperature, concentration of dissolved oxygen, COD in Utrata river, ranges of 1st and 2nd class of water quality according to Regulation of the Minister of the Environment of July 21 (2016)

The pH value was determined to be subject to considerable changes throughout the year. The lowest pH was recorded in October at control-measurement point 1 pH = 7.29, and the highest values were observed at point 2 in July pH = 8.08 (Fig. 2).

In all sampling terms, water showed higher pH values after leaving the reservoir. In the autumn and winter months (October to February), the pH value was within the ranges defined for water quality class I, while from March to September, the pH value exceeded the limits defined as water quality class II. Water temperature was strongly variable throughout the study period. The highest values were recorded in July (23 °C), and it was the only measurement that exceeded the threshold values of class I water quality. The lowest temperature values were measured in December and January and equalled 4 °C. The Komorów reservoir influenced the temperature reduction in the Urata River in the months of May, June, July, August, October and February. A temperature increase in the river below the reservoir occurred in December, January and March. Dissolved oxygen concentration was variable depending on the season and measurement point. The highest value of the index (11.94 mg O_2_ dm^−3^) was observed in January, at the measurement point behind the reservoir. The value did not exceed the threshold values for class I of water cleanliness. The lowest dissolved oxygen concentration was measured in August at the point in front of the reservoir (2.83 mg O_2_ dm^−3^). The value exceeded the threshold of class II of water quality. The concentration of oxygen in the Utrata River increased below the Komorów reservoir during each test period. The chemical oxygen demand, pointing to water pollution with organic substances, was strongly variable depending on the season and site. The lowest values were observed in April, and the strongest pollution was recorded in August, when the threshold value for class II of water cleanliness was exceeded. Except for the winter months (December–January), the concentration of the COD index was higher at the point below the Komorów reservoir.

**Fig. 3:**
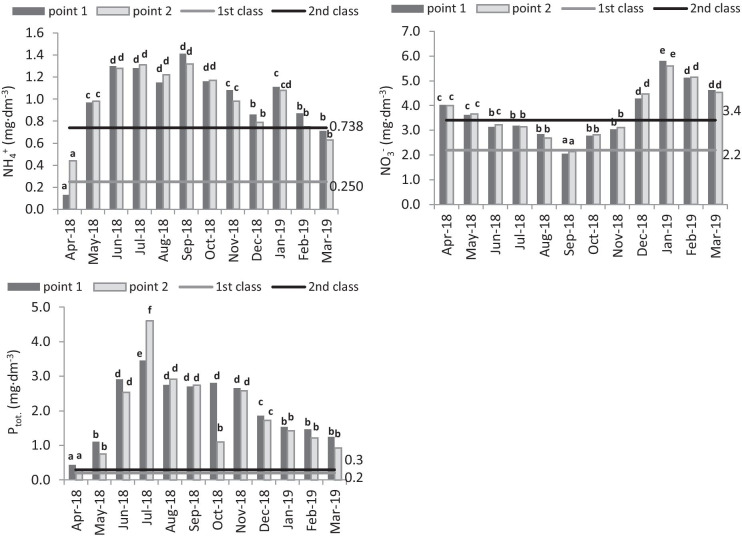
Seasonal variability of the of ammonium, nitrate (V) and total phosphorus concentration in the Utrata river, ranges of 1st and 2nd class of water quality according to Regulation of the Minister of the Environment of July 21 (2016)

Total phosphorus concentration was very high, and considerably exceeded the permissible threshold values for class II of water quality. The highest values were measured in summer months—4.6 mg P dm^−3^ in July—and the lowest in April—0.3 mg P dm^−3^ (Fig. 3). For the majority of the year, the reservoir contributed to a decrease in the quantity of pollutants flowing in the river. Only in summer months (July, August, September), the total phosphorus concentration was higher at the measurement point above, rather than below the reservoir. The concentration of ammonium nitrogen showed variability over the study period. The lowest values of the index occurred in spring months (April—0.13 mg N-NH_4_ dm^−3^) and the highest in summer and autumn months (September—1.41 mg N-NH_4_ dm^−3^) (Fig. 3). No significant influence of the retention reservoir of Komorów on ammonium nitrogen concentration in the Utrata River was observed. Ammonium nitrogen concentration decreased behind the reservoir in June, September, November, December, January, February and March; however, ammonium nitrogen concentration increased in April, May, July, August and October. The concentration of nitrate nitrogen was strongly variable over the study period. The lowest values were observed in September (point 1—2.05 mg N-NO_3_ dm^−3^; point 2—13 mg N-NO_3_ dm^−3^) and the highest in January (point 1—5.8 mg N-NO_3_ dm^−3^; point 2—5.6 mg N-NO_3_·dm^−3^). The Komorów Reservoir showed no considerable effect on changes in the concentration of nitrate and ammonia nitrogen in the Utrata river. In the winter months, the concentration of nitrate nitrogen was the highest, and the lowest concentration was observed in the autumn months.

## Discussion

In terms of the index, water in the Utrata River showed values exceeding those limits for class II of water quality. A general cause of higher than average pH can be sewage discharge to rivers, showing a particularly strong effect in warm months, when the water table is low. A cause of higher than average pH at point 2 as compared with point 1 can be development of algae reducing the content of CO_2_ in water. Similar pH results were observed by Adedeji et al., (2019) and Kuchko et al. (2020). Temperature below the reservoir in summer months was lower or equal to the value measured above the reservoir. Łaszewski et al. in 2018 showed that in the summer months, the temperature below the reservoir is higher than above the reservoir, which is at variance with the study results. It should be emphasised, however, that the Utrata in front of the reservoir in Komorów is not a regulated river. It flows slowly in a shallow channel, and therefore heats up much faster. Also, Jonczak and Parzych (2019), while studying Kamienna Stream, did not observe the influence of the reservoir on the water temperature in the river.

Throughout the study period, oxygen concentration was usually higher at the measurement point behind the Komorów Reservoir. Dissolved oxygen concentration in surface waters depends on many factors. It increases due to the process of photosynthesis and reaeration. A reduction of dissolved oxygen concentration can be caused by a higher than average temperature and mineralisation of organic substances (Rajwa-Kuligiewicz et al., 2015). In the case of the Utrata, the temperature at the point behind the reservoir is lower; this may increase the dissolved oxygen concentration. Another probable cause of the dissolved oxygen concentration increase is a dam situated at the river outlet, which causes mechanical aeration of the water. Ziułkiewicz (2019) also observed an increase in the dissolved oxygen concentration and chemical oxygen demand in the river after leaving the reservoir. An increase in the concentration of organic pollutants measured by the CODMn index was observed in the summer months. It may be associated with a higher than average quantity of pollutants of agricultural or municipal origin (Yu & Wu, 2018). The chemical oxygen demand value was always higher at the measurement point below the Komorów Reservoir. Notice the correlation, however, between the content of dissolved oxygen and value of chemical oxygen demand. When the content of the dissolved oxygen in water decreases, the chemical oxygen demand considerably increases, because of the lack of oxidant that would oxidise the reduced organic pollutants. Decreasing the speed of the water in the river flowing through the reservoir results in the sedimentation of nutrients at the bottom (Verstraeten & Poesen, 2000); however, in the case of the river Utrata, such dependence does not occur. This may be due to the high content of organic substances in the lake, which makes the sedimentation process difficult. A clear increase in the organic matter concentration at points below the reservoir can be observed at the end of the growing season, due to internal feeding from the bottom sediment of the Komorów reservoir. Due to its location, the Komorów reservoir may be exposed to the following sources of phosphorus: agriculture, urbanisation and sewage disposal. The causes of such high total phosphorus concentration are pollutants of agricultural and municipal origin (Wojtkowska & Bojanowski, 2018). Other sources of phosphorus in rivers can be the grazing of animals and unregulated sewage management (Ilić & Panjan, 2018). Organic pollutants flowing into the reservoir for many years have been deposited at the bottom, significantly increasing the potential of the internal charge (Qin et al., 2020). The presence of a weir in front of the discharge of water from the reservoir can disturb sediment deposition (Moore et al., 2012). Six kilometres above the reservoir, there is a sewage treatment plant in Walendow, which may affect the high concentration of ammonium nitrogen in the waters of the river Utrata before entering the Komorów reservoir. Another potential source of ammonium nitrogen is runoff from agricultural land and the decomposition of organic substances. In flowing waters, as a result of decomposition of organic substances, ammonia occurs. It is strongly bioavailable, contributing to the development of phytoplankton. Another source of ammonia nitrogen can be industrial and municipal sewage (Burzyńska, 2019). Measurements performed by the National Geological Institute determine ammonium nitrogen concentration in a range of 0.18–0.8 mg N-NH_4_ dm^−3^ in the Utrata river. Wiatkowski (2011a) evidenced that water reservoirs contribute to a decrease in the pollution of rivers flowing through them through the sedimentation process. In the case of the reservoir in Komorów, no such unambiguous dependency was determined, suggesting its high level of pollution. For most of the year, the studied reservoir reduced ammonium nitrogen concentration in the Utrata river, and a similar correlation was observed by Liu et al. (2019).

## Conclusion

The water quality in the Utrata River was the most negatively affected by nutrients, particularly by total phosphorus concentration. For the majority of the year, the Komorów Reservoir constituted no barrier for pollutants supplied through the Utrata River, as manifested in an increase in the value of CODMn and lack of N-NH_4_, N-NO_3_ concentration change in the river below the reservoir. The Komorów Reservoir positively affects the concentration of dissolved oxygen, decreases total P concentration and increases water pH and reduces the temperature in the summer months.
